# Allostatic load, a measure of cumulative physiological stress, impairs brain structure but not *β*-accumulation in older adults: an exploratory study

**DOI:** 10.3389/fnagi.2025.1508677

**Published:** 2025-03-31

**Authors:** Cassandre Palix, Léa Chauveau, Francesca Felisatti, Anne Chocat, Laurent Coulbault, Oriane Hébert, Florence Mézenge, Brigitte Landeau, Sacha Haudry, Séverine Fauvel, Fabienne Collette, Olga Klimecki, Natalie L. Marchant, Vincent De La Sayette, Denis Vivien, Gaël Chételat, Géraldine Poisnel, the Medit-Ageing Research Group

**Affiliations:** ^1^Normandie University, UNICAEN, INSERM, U1237, PhIND "Physiopathology and Imaging of Neurological Disorders", NeuroPresage Team, Cyceron, Caen, France; ^2^Department of Biochemistry, Caen Normandy Hospital (CHU de Caen), Caen, France; ^3^GIGA-CRC In Vivo Imaging and Psychology, Cognitive Neuroscience Research Unit, Liège University, Liège, Belgium; ^4^Deutsches Zentrum für Neurodegenerative Erkrankungen, DZNE, German Center for Neurodegenerative Disease, Dresden, Germany; ^5^Division of Psychiatry, University College London, London, United Kingdom; ^6^Department of Neurology, CHU de Caen, Caen, France; ^7^Department of Clinical Research, CHU de Caen, Caen, France

**Keywords:** allostatic load, aging, Alzheimer’s disease, neuroimaging, cognition, stress, brain, amyloid

## Abstract

**Introduction:**

Allostatic load (AL) is a composite score of progressive physiological dysregulations in response to long-term exposure to everyday stress. Despite growing interest, limited research has focused on links with cerebral and cognitive aspects of aging and with markers sensitive to Alzheimer’s disease (AD) in a healthy elderly population and with a multimodal approach.

**Methods:**

At baseline, 111 older adults (without cognitive impairment) from the Age-Well trial completed blood and anthropometric markers collection, cognitive assessments and multimodal neuroimaging within 3 months.

**Results:**

AL was negatively associated with gray matter volume and white matter integrity within frontal and temporal regions and poorer attentional performance.

**Discussion:**

AL is linked to structural brain integrity in aging- and stress-sensitive regions but not with AD-related markers (*β*-amyloid load) and only in two AD-sensitive brain regions in older adults. These results highlight the potential interest of AL as a sensitive index of stress-induced brain aging.

## 1 Introduction

The concept of Allostatic load (AL), initially proposed by McEwen and Stellar (1993), refers to the cumulative dysregulation across multiple physiological systems in response to long-term exposure to everyday stress or stress-related *wear and tear* on the body and brain (Seeman et al., 1997; Juster et al., 2010; McEwen, 1998). AL is a widely adopted model that captures the body’s adaptive response to stress, initiated by primary mediators such as stress hormones (from the neuroendocrine system) and cytokines (from the immune system). These mediators trigger cardiovascular, metabolic and second-order inflammatory changes aimed at maintaining stability, and referred as secondary outcomes (McEwen, 1998). While physiological systems are typically adaptive and resilient to short-term stress, chronic exposure to fluctuating responses due to repeated or prolonged stress can be deleterious, leading to physiological dysfunction and the development of stress-related diseases (Juster et al., 2010; McEwen, 1998). Numerous studies have expanded and refined the operationalization of AL, resulting in a composite score that encompasses the key physiological systems most affected by stress. Additionally, various computation methods for calculating AL have been developed, including count-based high-risk quartiles or z-score-based approaches (Carbone et al., 2022; Guidi et al., 2021; McLoughlin et al., 2020).

Growing evidence suggests that AL increases with age and is associated with poorer health outcomes (Read and Grundy, 2014; Seplaki et al., 2005). In later life, higher AL has been found to predict cardiovascular disease and is linked to earlier mortality (Booth et al., 2015; Seeman et al., 1997, 2001; Seplaki et al., 2005), as well as the development or worsening of various stress-related conditions (Guidi et al., 2021). Moreover, it has been proposed that the disruption of whole-body homeostasis and the resulting allostatic overload may progressively contribute to the development of Alzheimer’s disease (AD) (De Felice et al., 2022; Matos and De Souza-Talarico, 2019). However, the neurobiological mechanisms underlying this link remain unclear. To date, no study has examined the relationship between AL and brain structure, function, or *β*-amyloid deposition in healthy adults—key neuroimaging markers of aging and AD. Therefore, our aim was to further investigate the association of AL with cerebral *β*-amyloid deposition, as well as brain structure and function, to identify which brain regions—whether AD-sensitive or age-sensitive—are particularly vulnerable to elevated AL in older adults.

The brain’s central role as both a target of stress and a regulator of the body’s responses to stressors is evident in the long-term effects of physiological mediators involved in the stress response (Booth et al., 2015; McEwen and Gianaros, 2010). Brain regions including the hippocampus, amygdala, hypothalamus and prefrontal cortex undergo stress-induced structural remodeling, which alters behavioral and physiological responses (Arnsten, 2015; Arnsten et al., 2015; Marin et al., 2011; McEwen, 2007). High AL might be associated with various neurostructural and neurofunctional alterations in different populations (e.g., non-clinical, schizophrenia, overweight) (Lenart-Bugla et al., 2022). Few studies have explored the relationship between AL and stress-sensitive brain regions and the findings across studies have been mixed and sometimes contradictory. While one study found a positive association with hippocampal volume (Booth et al., 2015), another reported no significant relationship with hippocampal or prefrontal cortex volumes (Zsoldos et al., 2018). Higher AL was associated with lower gray matter (GM) volume in some studies (Ritchie et al., 2017; Zsoldos et al., 2018), but not in others (Booth et al., 2015), and with lower white matter (WM) volume (Booth et al., 2015; Ritchie et al., 2017). Additionally, while Ritchie et al. (2017) observed impaired WM integrity with higher AL, Zsoldos et al. (2018) found no such association. These discrepancies likely stem from significant heterogeneity across studies, including variations in the calculation of AL (e.g., differing numbers of biomarkers, ranging from 9 to 20, or the use of high-risk percentile vs. z-score methods) and the specific brain imaging parameters evaluated (Supplementary Table 1). Although stress is acknowledged as a contributor to brain aging, its exact impact on brain health remains uncertain, underscoring the need for further research.

Beyond the link with brain integrity, investigating the impact of elevated AL on cognitive abilities is particularly relevant in aging and stress-related impact on brain health. Indeed, much research has explored the association between chronic stress and the decline of specific cognitive functions, such as working and episodic memory, executive control, processing speed, and attention (Marin et al., 2011; Girotti et al., 2024). Chronic stress may disrupt attentional processes, which are integral to many other cognitive functions, thereby amplifying its broader impact on cognition (Liu et al., 2020). More specifically, a chronic exposure to stress hormones such as cortisol has been associated with impaired cognitive performance, particularly on hippocampal-dependent memory tasks (Marin et al., 2011; Seeman, 1997). Several studies have investigated the association between AL and cognition in older adults (Booth et al., 2015; Juster et al., 2010; Karlamangla et al., 2002; Narbutas et al., 2019, 2021; Seeman et al., 2001) and highlighted that higher AL was related to lower global cognition and executive functions, but not working and episodic memory in healthy younger and older adults (D’Amico et al., 2020).

Although a substantial body of research has demonstrated associations between individual AL components—such as cortisol (Lupien et al., 2009; Sapolsky et al., 2000), heart rate variability (Mather and Thayer, 2018), glycaemia (Palix et al., 2022) and waist-to-hip ratio (Ronan et al., 2016; Smith et al., 2011)—and brain structure or cognitive function (Juster et al., 2010), relatively few studies have explored these biomarkers collectively. The utility of an aggregate AL score lies in its ability to capture the cumulative burden of chronic stress on multiple physiological systems, providing a more comprehensive measure of stress-related dysregulation than any single biomarker alone (McEwen and Stellar, 1993; Karlamangla et al., 2006; Schulkin, 2004; Beckie, 2012). By examining AL as a multidimensional construct, this study aims to elucidate its broader impact on brain structure and cognitive functioning, beyond the effects of individual biomarkers.

Despite the brain’s central role in the stress response and the well-documented impact of chronic stress on brain health, research on the effects of AL remains limited. Moreover, findings are inconsistent, with highly variable and often unreliable methods used to measure AL. Further studies are needed to improve our understanding of the relationship between AL and the cerebral and cognitive aspects of aging, as well as with the early manifestations of AD reflected by AD biomarkers. To meet this need, we assessed, in a cross-sectional study, the links between AL, calculated using a reliable method including 18 biomarkers, and a wide array of complementary neuroimaging and cognitive function measures, in the same population of well-selected healthy older adults. We hypothesized that higher AL scores would be associated with (i) poorer brain integrity specifically in stress-sensitive brain regions, (ii) the presence of AD biomarkers, (iii) and lower cognitive functioning.

## 2 Materials and methods

### 2.1 Participants

**Figure 1:**
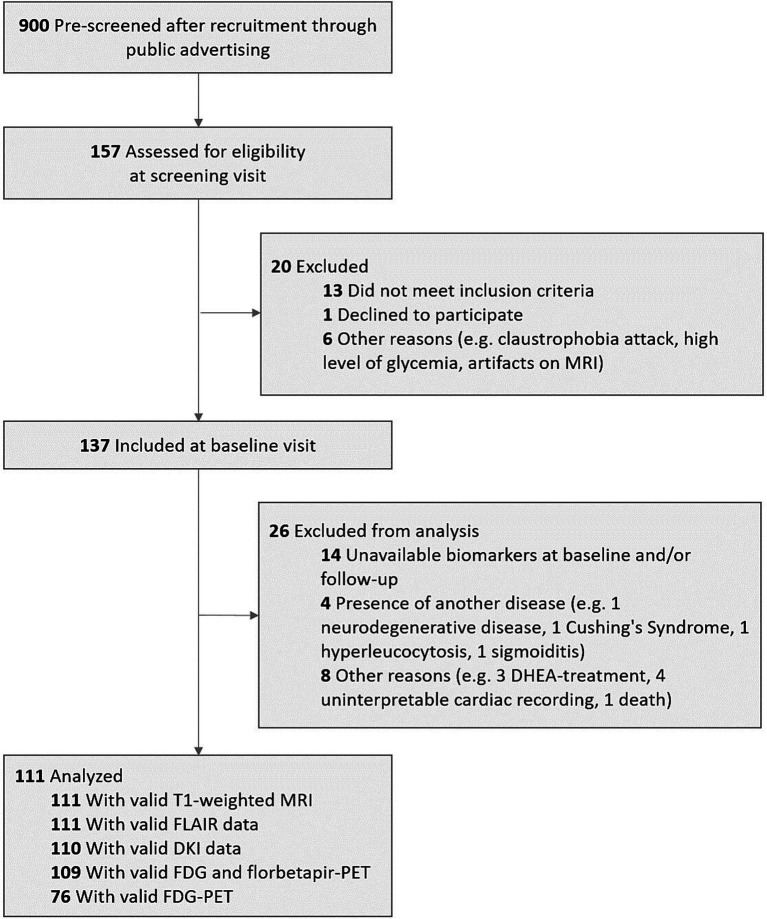
Flow diagram of the inclusion process. DHEA, dehydroepiandrosterone; DKI, Diffusion Kurtosis imaging; FDG, 18F-fluorodeoxyglucose; MRI, magnetic resonance imaging; PET, positron emission tomography.

We included 111 older adults from the baseline visit of the Age-Well randomized clinical trial of the Medit-Ageing European Project (NCT02977819) (Poisnel et al., 2018) (flow diagram in Figure 1), sponsored by the French National Institute of Health and Medical Research (INSERM). Participants were recruited from the general population from November 2016 until March 2018. They were all native French speakers, aged over 65 years, educated for at least 7 years and cognitively unimpaired, having performed within the normal range for age and educational level on standardized cognitive tests [Global Cognitive Functioning: Mini-Mental State Examination (MMSE) (Folstein et al., 1975); Executive Functions: Modified Card Sorting Test (Godefroy O et le GRoupe de Réflexion sur l’Evaluation des Fonctions EXécutives, 2008); Verbal Episodic Memory: RL-RI16 test (Van der Linden et al., 2004); Depressive State: Montgomery and Asberg Depression Rating Scale (Montgomery and Asberg, 1979); English Proficiency: Test of Written and Oral Comprehension]. The main exclusion criteria were evidence of major neurological or psychiatric disorders (including an addiction to alcohol or drugs), history of cerebrovascular disease, chronic disease or acute unstable illness (respiratory, cardiovascular, digestive, renal, metabolic, hematologic, endocrine or infectious) and current medication that may interfere with cognitive functioning (psychotropic, antihistaminic with anticholinergic action, anti-Parkinson’s, benzodiazepines, steroidal anti-inflammatory long-term treatment, antiepileptic or analgesic drugs) (Poisnel et al., 2018). The full inclusion and exclusion criteria, for the Age-Well trial and the analyses, are detailed in the Supplementary Methods 4. Participants meeting inclusion criteria underwent a detailed cognitive and psychologic assessment and blood tests, structural MRI, ^18^F-fluorodesoxyglucose (FDG)- and AV45-PET scans within a maximum of 3 months. Baseline data were collected from November 2016 until April 2018. All participants gave their written informed consent to participate in the study. The Age-Well randomized clinical trial was approved by the ethics committee (Comité de Protection des Personnes Nord-Ouest III, Caen, France; trial registration number: EudraCT: 2016–002441-36; IDRCB: 2016-A01767-44; ClinicalTrials.gov Identifier: NCT02977819). Participant characteristics are presented in Table 1.

**Table 1:** Participant characteristics.

Characteristics	Mean (SD)	Range
*N*	111	
Age, years	68.88 (3.82)	65–83
Female, no. (%)	70 (63%)	
Education, years	13.23 (3.19)	7–22
Global *β*-amyloid deposition (SUVr)	1.2 (0.13)	0.97–1.70
*β*-amyloid status no. (%)^a^	41 (37%)	
Current smoking status no. (%)	6 (5%)	
Current medication use no. (%)	51 (46%)	
Antihypertensive treatment no. (%)	39 (35%)	
Cardiovascular treatment no. (%)	42 (38%)	
Antidiabetic treatment no. (%)	2 (2%)	
Hypoglycemic treatment no. (%)	2 (2%)	
Hypocholesterolemic treatment no. (%)	19 (17%)	
Anti-inflammatory treatment no. (%)	2 (2%)	
**Allostatic load**		
*Anthropometric category*		
BMI, kg/m^2^	26.34 (4.41)	18.39–44.18
WHR, cm/cm	0.91 (4.08)	0.71–1.15
*Cardiovascular and respiratory category*		
SBP, mmHg	134.80 (2.48)	87.7–198
DBP, mmHg	80.00 (10.02)	57.67–107
Pulse pressure, mmHg	54.80 (15.81)	17.70–96.30
RMSSD, ms	30.34 (38.85)	4.50–270.95
SDNN, ms	30.18 (26.21)	7.26–194.92
*Metabolic category*		
Creatinine, μmol/L	72.25 (11.48)	51–104
Triglycerides, mmol/L	1.24 (0.46)	0.62–3.04
HDL, mmol/L	1.70 (0.39)	0.97–3.08
LDL, mmol/L	4.06 (0.98)	1.71–7.82
Insulin, pmol/L	64.23 (31.02)	17–177
*Immune category*		
hsCRP, pg/mL	2.74 (4.33)	0.16–31.37
IL-6, pg/mL	0.87 (1.06)	0.06–8.52
*Neuroendocrine category*		
DHEA-S, μmol/L	2.55 (1.65)	0.20–8.70
Cortisol, nmol/L	439.19 (99.79)	233–736
Epinephrin, μg/L	0.04 (0.03)	0.01–0.16
Norepinephrin, μg/L	0.59 (0.22)	0.22–1.24
**Cognition composite scores**		
Global cognition (PACC-5)	−0.03 (1.01)	−3.01-1.14
Processing speed composite	0.03 (0.72)	−2.37-1.48
Selective attention composite	0.01 (0.74)	−2.30-2.09
Executive function composite	−0.05 (0.68)	−2.07-1.44
Episodic memory composite	0.03 (0.96)	−2.81-1.93
**Global neuroimaging values**		
Gray matter volume (mm^3^)	608,312 (27,488)	515,984–670,626
Glucose metabolism	1.10 (0.06)	0.98–1.25
Brain perfusion (SUVr)	0.94 (0.39)	0.84–1.04
White matter hyperintensities	4.02 (5.26)	0.09–36.55
White matter—mean kurtosis	0.96 (0.04)	0.83–1.12
White matter—fractional anisotropy	0.29 (0.02)	0.24–0.35

BMI, body mass index; WHR, waist-hip-ratio; SBP and DBP, systolic and diastolic blood pressure; RMSSD, root mean square of successive differences between normal heartbeats; SDNN, standard deviation of the average heart beat-to-beat intervals; HDL and LDL, high and low density lipoprotein; hsCRP, high sensibility C-reactive protein; IL-6, interleukin-6; DHEA-S, dehydroepiandrosterone sulfate; SUVR, standard uptake value ratio; PACC-5, Preclinical Alzheimer’s Cognitive Composite 5. ^a^*β*-amyloid status no. (%) refers to the proportion of participants whose neocortical *β*-amyloid deposition fell above the threshold for *β*-amyloid positivity. This threshold corresponded to the 99.9th percentile of the neocortical standard uptake value ratio distribution among 45 healthy young individuals younger than 40 years (see details in André et al., 2020).

### 2.2 Allostatic load

AL was calculated using 18 different biomarkers associated with neuroendocrine, immune, metabolic, cardiorespiratory and anthropometric categories. *Neuroendocrine* biomarkers included (1) integrated measures of HPA axis functioning: serum cortisol and dehydroepiandrosterone sulfate (DHEA-S) levels, and (2) measures of sympathetic nervous system activity: plasma epinephrine and norepinephrine levels. *Immune* biomarkers included systemic markers of inflammation: plasma interleukine-6 and high-sensitivity C-reactive protein. *Metabolic* biomarkers included (1) plasma insulin, a measure of glucose metabolism regulation, (2) serum triglycerides, high-density lipoprotein (HDL) and low-density lipoprotein (LDL) cholesterol levels, to assess lipid metabolism, and (3) serum creatinine, a measure of kidney function, muscle and protein metabolism. *Cardiovascular and respiratory* biomarkers included (1) systolic, diastolic blood pressure and pulse pressure, to assess cardiovascular functioning and (2) two electrocardiogram measures, a standard deviation of the average heart beat-to-beat intervals (SDNN), and root mean square of successive differences between normal heartbeats (RMSSD), to assess resting heart rate variability and parasympathetic nervous system functioning. *Anthropometric* biomarkers included (1) waist-to-hip ratio, a measure of adipose tissue deposition and (2) body mass index, a measure of body fat. We selected the biomarkers to include in the AL measure based on the review by Kallen et al. (2021), which provides a comprehensive analysis of AL biomarkers, emphasizing those with significant variability and relevance in aging populations and offering a valuable framework for assessing cumulative physiological stress and supporting healthy aging interventions (Kallen et al., 2021 and Supplementary Methods 1 for detailed biological measures).

To ensure the robustness of our analyses, we assessed the skewness of all biomarkers and applied appropriate transformations, such as log transformations, to those with significantly non-normal distributions. Outliers were identified using the Grubbs test and their impact was evaluated by conducting analyses both with and without these values. As no significant impact on the results was observed, outliers were retained to preserve sample size.

Each of the five categories (i.e., neuroendocrine, immune, metabolic, cardiorespiratory, and anthropometric) was z-transformed and averaged. Before averaging, z-scores derived from HDL, DHEA-S, SDNN and RMSSD were reversed so that increasing values always indicated poorer health. AL was calculated as the z-score of the average of the 5 categories. This scoring allowed us to create an AL score that retained the continuous properties of each physiological variable (McLoughlin et al., 2020; Narbutas et al., 2019). Higher values of AL indicate higher multi-system physiological dysregulation.

### 2.3 Neuroimaging procedure

Participants underwent a structural T1, FLAIR and Diffusion Kurtosis imaging (DKI) MRI, as well as 18F-fluorodeoxyglucose (FDG)- and AV45-PET scans, to measure GM volume, WM hyperintensities and integrity [i.e., mean diffusivity (MD) and fractional anisotropy (FA)], brain glucose metabolism, brain perfusion and *β*-amyloid burden. All examinations were performed at the Cyceron Center (Caen, France). Details of MRI and PET images acquisition and processing are available in previous publications (Ourry et al., 2021; Tomadesso et al., 2018) and are described in Supplementary Methods 2. Briefly, T1-weighted images were processed in SPM12. FLAIR images were segmented by the lesion prediction algorithm LPA (Schmidt, 2016) in SPM12 and corrected using a specific corticospinal tract mask described elsewhere (Garnier-Crussard et al., 2020). DKI images were processed using MRI data for spatial normalization and diffusional kurtosis estimator software for extraction. FDG- and AV45- PET images were processed using MRI data for spatial normalization. The images resulting from the preprocessing, described in Supplementary Methods 2, were used to extract global neuroimaging values and were then smoothed for the voxel-wise analyses. Average global neuroimaging values were obtained using a binary mask of GM for preprocessed MRI and PET images and of WM for preprocessed DKI images.

### 2.4 Neuropsychological examination

From an extensive battery of cognitive tests, composite scores for the following domains were created: global cognition (Preclinical Alzheimer’s Cognitive Composite 5, PACC5) with Mattis Dementia Rating Scale-2 (Global score), Long-term free recall from the California Verbal Learning Test, second edition (CVLT-II), Digit Symbol Substitution Test from the WAIS IV (Raw note), Long-term free recall from the Logical Memory Test (Story B) from the WMS IV, Category Fluency (number of correct Animals recalled in 2 min); processing speed composite score with TMT A (time to perform the Trail Making Test part A), Stroop Test, reading time (time to complete the word card, reading condition), Stroop Test, naming time (time to complete the color card, naming condition); selective attention composite score with Digit Symbol Substitution Test from the WAIS IV (Raw note), Number of correct items at the D2-R Test, Percentage of errors at the D2-R Test; executive functions composite score with Digit Span Backward from the WAIS IV (Raw note), TMT B (time to perform the Trail Making Test part B), Stroop Test, interference index (time difference between the interference and naming conditions), Verbal Fluency (number of correct P-words recalled in 2 min) and episodic memory composite score with the sum of trials 1–5 from CVLT-II, Short-term free recall from CVLT-II, Long-term free recall from CVLT-II, Short-term recall from Logical Memory (Story B), WMS IV, Long-term recall from the Logical Memory (Story B), WMS IV (Supplementary Methods 3).

### 2.5 Statistical analysis

First, a partial correlation analysis was performed to assess the relationship between age and AL, and education and AL in the whole sample. Difference in AL between men and women was also assessed using t tests. All analysis were adjusted for age, sex, education, smoking status, and current medication, as these factors can influence the physiological biomarkers included in the AL score (e.g., smoking status and cortisol levels, or antihypertensive treatment and cardiovascular biomarkers).

To assess whether there was a specific association between AL and (1) each neuroimaging modality, as well as (2) cognitive scores, multilinear regressions were performed using each global neuroimaging value or cognitive scores as a dependent variable, AL as an independent variable and demographics (i.e., age, sex, education) and smoking status and current medication use as covariates. Finally, when a significant link was found between AL and the global value for a specific neuroimaging modality, the corresponding analysis was repeated voxelwise using SPM12, controlling for the same covariates, to further assess the regional specificity of the association. Voxelwise analyses were considered significant at a voxel-level threshold of *p* < 0.001 and a cluster-level threshold of *p* < 0.05, corrected for familywise errors (FWE).

Results were considered significant at a *p* < 0.05 threshold, then Bonferroni correction was applied to control for multiple comparisons so that results surviving a *p ≤* (0.05/number of comparisons) were indicated.

## 3 Results

### 3.1 Effect of demographics on AL

A negative association was found between education level and AL such as higher education was related to lower AL (Pearson *r* = −0.208 [95% CI, −0.127; −0.004]; *p =* 0.036). No association was found between age and AL (Pearson *r* = −0.016 [95% CI, −0.057–0.049]; *p* = 0.874). Finally, AL was higher in men than in women (*p* = 0.007; partial *η*^2^ = 0.068; power = 0.793).

### 3.2 Associations between AL and brain integrity

The results of the multiple linear regressions between AL and global neuroimaging values are presented in Table 2. No association was found between brain glucose metabolism, brain perfusion, *β*-amyloid deposition, or WM hyperintensities and AL. Negative associations were found between AL and both GM volume and WM FA. WM MD was positively associated with AL. Only the association between AL and GM volume survived the Bonferroni correction (*p =* 0.00714; 0.05/7).

**Table 2:** Multiple linear regressions between AL and global neuroimaging values adjusted for age, sex, education, smoking status and current medication use.

	Allostatic load (unstandardized *β*, [95% CI], *p*-value)
Global neuroimaging values	
Gray matter volume^1^	***β*, −0.009 [95% CI, −1.69e** ^**−5**^ **; −2.71e** ^**−6**^ **], *p* = 0.007**
Gray matter glucose metabolism^1^	*β*, 0.072 [95% CI, −0.012; 0.023], *p* = 0.54
Gray matter brain perfusion^1^	*β*, −0.080 [95% CI, −6.93; 2.79], *p* = 0.40
*β*-amyloid deposition^1^	*β*, −. 029 [95% CI, −1.64; 1.19], *p* = 0.75
White matter hyperintensities	*β*, 0.051 [95% CI, −0.03; 0.05], *p* = 0.64
White matter MD^2^	*β*, 0.352 [95% CI, 0.53; 6.86], *p* = 0.023
White matter FA^2^	*β*, −0.199 [95% CI, −20.20; −0.77], *p* = 0.03

FA, fractional anisotropy; MD, mean diffusivity. ^1^In a mask of total GM including voxels with a GM probability > 60% and excluding the cerebellum; ^2^In a mask of total WM including voxels with a WM probability > 60% and excluding the cerebellum. Bonferroni correction was applied to control for multiple comparisons so that a threshold of *p* < 0.00714 (0.05/7) was required for results to be considered significant (in bold).

**Figure 2:**
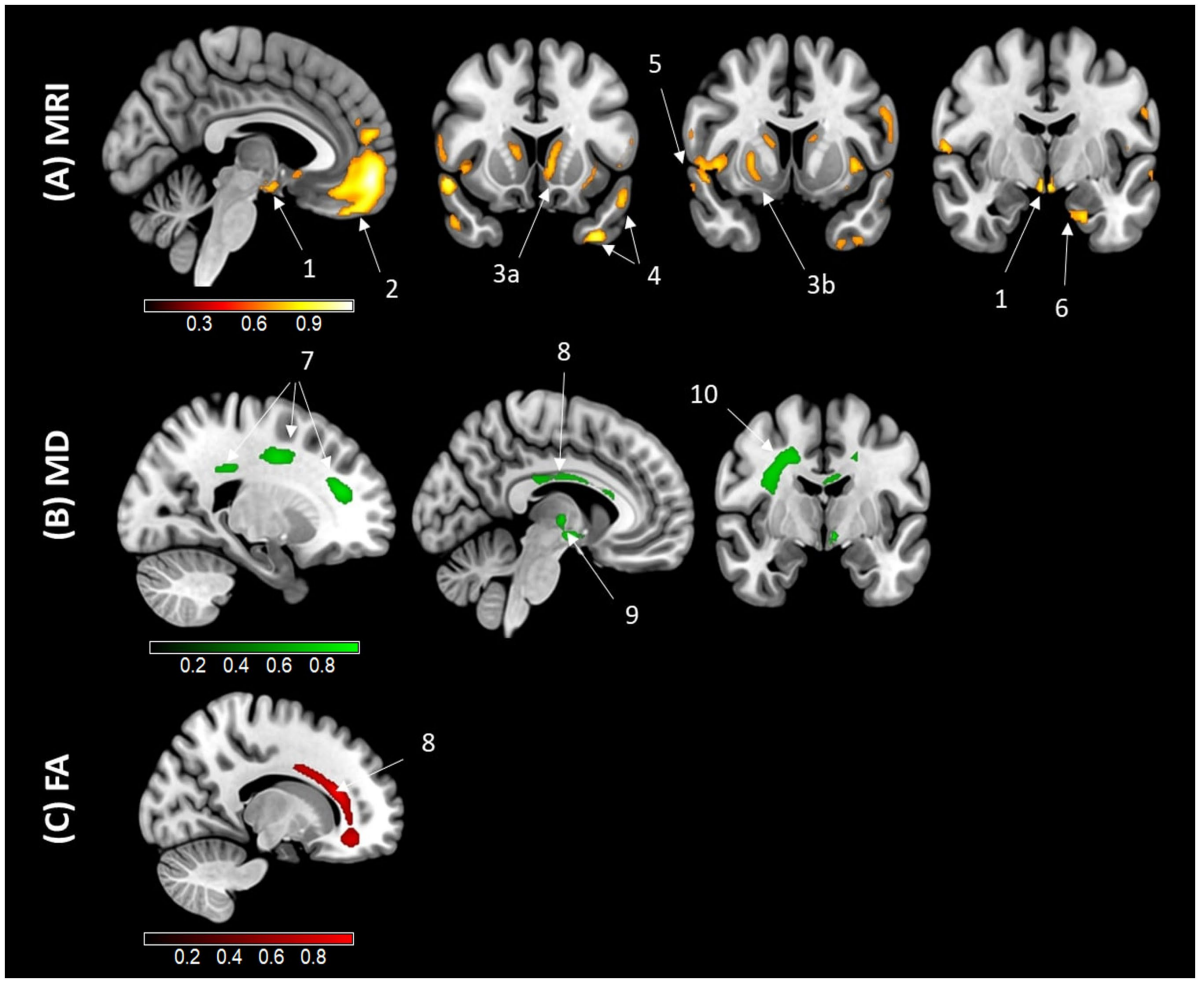
Results of the voxelwise correlation analyses illustrating the regional associations with AL. Results of the voxelwise analyses exhibit an association between higher AL and lower gray matter (GM) volume **(A)**, higher white matter mean diffusivity (MD) **(B)**, and lower white matter fractional anisotropy (FA) **(C)**, using magnetic resonance imaging (MRI) in older adults. Analyses were adjusted for age, sex, education, smoking status, and current medication use. Results were obtained at a *p* < 0.001 (uncorrected) threshold, and only clusters surviving a familywise error–cluster-level correction are reported. (1) Hypothalamus (x = 88, y = 123, z = 60); (2) Orbitofrontal and prefrontal cortex (x = 88, y = 173, z = 61); (3) Basal ganglia (a. Caudate (x = 82, y = 141, z = 76); b. Putamen (x = 196, y = 236, z = 132)); (4) Temporal gyrus (a. (x = 55, y = 141, z = 32); b. (x = 41, y = 141, z = 53)); (5) Insular gyrus (x = 258, y = 236, z = 121); (6) Parahippocampal gyrus (x = 113, y = 196, z = 82); (7) Corona radiata (a. (x = 198, y = 137, z = 207); b. (x = 198, y = 211, z = 218); c. (x = 198, y = 239, z = 174)); (8) Corpus callosum ((MD) (x = 137, y = 169, z = 193); (FA) (x = 186, y = 274, z = 175)); (9) Thalamic radiation (x = 137, y = 201, z = 127); (10) Longitudinal fasciculus (x = 208, y = 201, z = 205). Areas in yellow and red indicate regions negatively associated with AL, while areas in green indicate regions positively associated with AL.

Voxelwise analyses were conducted to assess the regional specificity of the relationships between AL and GM volume, WM MD and WM FA. The results are presented in Figure 2. A negative association was observed between AL and GM volumes in the prefrontal and orbitofrontal cortex, parahippocampal cortex, hypothalamus, mammillary bodies, temporal and insular cortex, as well as the basal ganglia (specifically the caudate and putamen nuclei). In terms of WM integrity, AL was positively associated with the corona radiata, corpus callosum, longitudinal fasciculus, and thalamic radiation. Lastly, a negative association between AL and WM FA was identified exclusively in the corpus callosum.

### 3.3 Associations between AL and cognitive functioning

A positive relationship was found between AL and the selective attention composite score, indicating that a higher AL score was associated with poorer attentional performance (since a higher selective attention composite score reflects lower attentional performance). This latter survived the Bonferroni correction for multiple comparisons. No association was found between global cognition, processing speed, executive functions or episodic memory composite scores and AL. Associations between AL and cognitive composites are presented in Table 3.

**Table 3:** Multiple linear regressions between AL and cognitive composite scores adjusted for age, sex, education, smoking status, and current medication use.

	Allostatic load (unstandardized *β*, [95% CI], *p-*value)
Cognitive composite scores	
PACC-5	*β*, −0.105 [95% CI, −0.22; 0.21], *p* = 0.96
Processing speed	*β*, 0.063 [95% CI, −0.17; 0.35], *p* = 0.49
Selective attention	***β*, 0.222 [95% CI, 0.16; 0.69], *p* = 0.002**
Executive functions	*β*, −0.210 [95% CI, −0.55; 0.04], *p* = 0.09
Episodic memory	*β*, −0.090 [95% CI, −0.22; 0.21], *p* = 0.98

PACC-5, Preclinical Alzheimer’s Cognitive Composite 5. Bonferroni correction was applied to control for multiple comparisons so that a threshold of *p* < 0.01 (0.05/5) was required for results to be considered significant (in bold).

### 3.4 Complementary analyses

In order to identify whether and which specific categories of AL were more strongly associated with brain and cognitive measures, we reran our analyses for each category separately (i.e., neuroendocrine, immune, metabolic, cardiorespiratory and anthropometric categories).

The associations between global neuroimaging measures, cognitive composite scores, and each AL category are presented in the Supplementary Tables 2, 3. In the adjusted model, no associations were found between the neuroendocrine, immune, metabolic, or cardiorespiratory categories and global neuroimaging values. However, the anthropometric category was negatively associated with GM volume and positively associated with WM MD, though not with other global neuroimaging measures (Supplementary Table 2).

In terms of cognition, no associations were observed between the neuroendocrine, metabolic, or anthropometric categories and cognitive composite scores in the adjusted model. The immune category, however, was negatively associated with the executive function composite score, but no associations were found with the other cognitive composite scores (Supplementary Table 3).

Additionally, to better understand the potential influence of primary mediators (i.e., neuroendocrine and immune categories) on other physiological systems (metabolic, cardiorespiratory and anthropometric categories), associations between primary mediators and other AL categories were performed. The immune category, however, was negatively associated with the metabolic category, but no associations were found with the other categories (surviving the Bonferroni correction). Results are presented in Supplementary Table 4.

## 4 Discussion

The main goal of the present study was to provide a comprehensive overview of the associations between AL and brain integrity in older adults. Our results showed that AL was negatively associated with GM volume and WM integrity in frontal and temporal regions but not with functional integrity (brain metabolism and perfusion), *β*-amyloid deposition, or WM hyperintensities. Interestingly, higher AL was also associated with poorer selective attentional performance.

We found that AL was higher in men than in women and in participants with lower education, which is consistent with most previous studies (Juster et al., 2016; Kerr et al., 2020; Langellier et al., 2021; Tampubolon and Maharani, 2018; Upchurch et al., 2015). In contrast, we found no association between AL and age. This contrasts with previous studies that reported that AL increases with age (Guidi et al., 2021; Moore et al., 2021). This discrepancy likely reflects the fact that our population had a limited age range (65–85 years) compared to previous studies (i.e., 18–80 years) (Guidi et al., 2021; Langellier et al., 2021; Moore et al., 2021; Tampubolon and Maharani, 2018; Upchurch et al., 2015).

Our results highlighted a significant link between elevated AL and lower GM volume and WM integrity. Only three previous studies investigated the relationship between AL and brain structure in older adults, and results were heterogeneous. One study did not find a link with GM volume (Booth et al., 2015) whereas the other two found a negative association with GM volume (Ritchie et al., 2017; Zsoldos et al., 2018) but among these two studies, only one found a link with WM integrity (Ritchie et al., 2017). These heterogeneous results may be explained by methodological differences as these previous studies only included 10 or fewer biomarkers in the AL score and did not use stress-related markers (Supplementary Table 1 for detailed differences). In the present study, with an AL score including 18 biomarkers relevant in aging population and including stress-related markers (Kallen et al., 2021), we showed a link between AL and brain structural integrity. In addition, we found that AL was mainly associated with GM volume in the insular cortex, frontal regions (prefrontal cortex and orbitofrontal regions), basal ganglia (caudate and putamen nuclei) and lateral and medial temporal regions (parahippocampal cortex, superior and inferior temporal cortex) and WM integrity in the corpus callosum, corona radiata, thalamic radiation and longitudinal fasciculus. Some of these regions were also found in previous studies - including the insular cortex, superior temporal gyrus and temporal gyrus (Zsoldos et al., 2018). In contrast, no previous study found a link between AL and frontal regions, basal ganglia and WM microstructures. Interestingly, we found that higher AL was related to lower GM volume in prefrontal cortex and hypothalamus, two stress-sensitive brain regions, but not in hippocampus or amygdala. This fronto-temporal pattern of atrophy and the WM microstructures associated with AL encompass brain regions that are sensitive to both normal aging (Bendlin et al., 2010; Fjell et al., 2014), and chronic stress (Arnsten, 2000; Arnsten et al., 2015; Saeedi and Rashidy-Pour, 2021; Stomby et al., 2016). These findings reinforce the notion that AL may serve as a marker of stress-related brain aging.

To our knowledge, our study is the first to investigate the association between AL and functional (i.e., glucose metabolism and perfusion), or molecular (i.e., *β*-amyloid accumulation) neuroimaging markers. We found that glucose metabolism and perfusion, WM hyperintensities and *β*-amyloid accumulation were not associated with AL in healthy older adults. While AL is not associated with AD-related neuroimaging markers, such as *β*-amyloid accumulation, two brain regions sensitive to AD—specifically the inferior temporal cortex and parahippocampus—were found to be related to AL. Although chronic stress and dysfunctions of the stress system have been previously related to the pathophysiology of AD and have been associated with increased risk of AD (Ennis et al., 2017; Prenderville et al., 2015; Saeedi and Rashidy-Pour, 2021), we did not clearly find this relationship in our population at an asymptomatic stage. Further investigation involving patients along the AD clinical continuum—from subjective cognitive decline to mild cognitive impairment and AD—would provide valuable insights into the potential role of AL in dementia.

We did not find an association between AL and global cognition, executive functions, or processing speed and episodic memory, counter to the findings synthesized in a meta-analysis (D’Amico et al., 2020). This may be explained by the relatively small sample size (i.e., 111 individuals) and age-range of our population (i.e., 65–85 years) compared to studies examined in the meta-analysis (i.e., each study included more than 600 individuals aged 20–74 years or above 50 years only) (D’Amico et al., 2020). However, to the best of our knowledge, our results provide clear evidence that, in older adults, higher AL is associated with reduced selective attentional capacity. This is particularly relevant considering that various aspects of attention, along with other cognitive functions, have been shown to decline with aging (Commodari and Guarnera, 2008; McAvinue et al., 2012).

Furthermore, our complementary analyses to explore the relationships between separate AL categories and the outcome variables, showed that the anthropometric category was significantly associated with brain structural integrity (i.e., GM volume and WM MD) and that the immune category was associated with executive functions. Overall, we did not find significant associations between primary mediators and the other AL categories, except for a link between the neuroendocrine and metabolic categories. While our findings do not fully confirm the theoretical sequence connecting primary stress mediators (neuroendocrine and immune markers) to secondary outcomes (such as metabolic, cardiorespiratory, and anthropometric markers), they do partially align with this framework (Juster and Misiak, 2023). Additionally, our results suggest that the associations found here are not solely driven by primary mediators, highlighting a complex interplay among physiological systems. This reinforces the value of integrating multiple biomarkers into an index to better capture the relationship with stress-related changes in brain function and cognition.

The current findings provide a valuable contribution to our understanding of the damaging effects of AL on brain integrity during aging. Future studies with larger and more diverse samples, spanning the entire continuum, from normal cognition to AD dementia, are needed to unravel the association between AL, its individual components and brain health, investigate underlying mechanisms, and explore group-specific associations through stratified analyses that account for variations in age, sex, and educational backgrounds.

Our study has several limitations. First, while chronic diseases were an exclusion criterion, participants with diabetes, prediabetes, and obesity were included if their treatments were stabilized and well-balanced. Individuals with a glycemia level higher than 8.89 mmol/L, indicating prediabetes or untreated diabetes, were excluded. These conditions are associated with various biomarkers, including blood pressure, cortisol, and cholesterol levels (Abdulraheem Jabbar and Jalal Majeed, 2020; Fraser et al., 1999; Salehi et al., 2019). Previous studies have indicated that AL negatively correlates with GM volume and WM integrity, particularly in overweight adults, suggesting that obesity may influence the relationship between AL and brain integrity (Ottino-González et al., 2018; Ottino-González et al., 2017). Second, cortisol measurements were obtained from a single blood sample taken in the morning at the same time. Given that cortisol follows a circadian rhythm, previous studies have recommended collecting samples at multiple times throughout the day to better capture overall cortisol levels (El-Farhan et al., 2017). Third, despite efforts to establish a consensual definition, AL remains a concept to be clarified, whose validity is still debated, as pointed out by McCrory et al. (2023), who stress the difficulties linked to the precision of its measurement and its interpretation from one study to another. Fourth, while the sample distribution is adequate, the relatively small sample size, combined with the limited range of *β*-amyloid load and the low proportion of *β*-amyloid-positive participants in this cognitively normal sample, may have constrained our ability to detect subtle effects across certain dimensions, such as age and the relationship between AL and *β*-amyloid accumulation. Finally, the cross-sectional design of our analyses limits the interpretation of our findings; longitudinal studies are needed to investigate the relationships between AL and brain integrity over time and to determine whether these changes are reversible.

## 5 Conclusion

The association between AL and brain integrity identified in this study underscores AL’s relevance to structural brain health in older adults. The fronto-temporal brain regions and the WM microstructures linked to elevated AL are primarily sensitive to aging and stress. Furthermore, the negative association between AL and selective attention performance—which is known to share neural networks with executive functions, especially in the prefrontal cortex (Arnsten, 2015) and to be affected by both aging and stress—strengthens this interpretation. Notably, we have also demonstrated for the first time that higher AL is not associated with *β*-amyloid deposition, but rather with several AD-sensitive brain regions. Thus, while AL appears to be a leading measure of stress-induced brain aging, its relationship with AD risk warrants further investigation.

## Data Availability

Data and code are made available on request following a formal data sharing agreement and approval by the consortium and executive committee (https://silversantestudy.eu/2020/09/25/data-sharing). Data sharing policies are in compliance with our ethics approval and guidelines from our funding body (https://silversantestudy.eu/2020/10/01/silver-sante-study-data-sharing-now-available).

## Group members of the Medit-Ageing Research Group

Florence Allais^8^, Claire André^1^, PhD, Sebastian Baez Lugo^4,9^, MSc, Pierre Champetier^1^, MSc, Chauveau Léa^1^, MSc, Gaël Chételat^1^, PhD, Anne Chocat^1^, MD, Fabienne Collette^3^, PhD, Sebastien Czajko^10^, MSc, Sophie Dautricourt^1^, MD, PhD, Robin De Flores^1^, PhD, Marion Delarue^1^, MSc, Vincent De La Sayette^6^, MD, PhD, Rawda El Sadawy^1^, MSc, Séverine Fauvel^1^, BA, Francesca Felisatti^1^, MSc, Victor Ferment^1^, BA, Eglantine Ferrand Devouge^1^, MD, Eric Frison^8^, MD, Antoine Garnier-Crussard^10^, MD, MSc, Julie Gonneaud^1^, PhD, Anaïs Hamel^1^, MSc, Sacha Haudry^1^, MSc, Oriane Hébert^1^, MSc, Marc Heidmann^10^, MSc, Olga Klimecki^4^, PhD, Elizabeth Kuhn^1^, PhD, Brigitte Landeau^1^, MSc, Gwendoline Ledu^1^, MSc, Valérie Lefranc^1^, Natalie Le Marchant^5^, PhD Antoine Lutz^10^, PhD, Florence Mezenge^1^, BA, Cassandre Palix^1^, MSc, Léo Paly^1^, MSc, Valentin Ourry^1^, PhD, Géraldine Poisnel^1^, PhD, Anne Quillard^1^, MD, Géraldine Rauchs^1^, PhD, Stéphane Rehel^1^, PhD, Florence Requier^3^, MSc, Eric Salmon^3^, MD, PhD, Clémence Tomadesso^1^, PhD, Edelweiss Touron^1^, PhD, Anne-Laure Turpin^1^, MSc, Denis Vivien^1,7^, PhD, Patrik Vuilleumier^4^, MD, and Cédrik Wallet^8^, PhD, Miranka Wirth^11^, PhD.

^1^Normandie Univ, UNICAEN, INSERM, U1237, PhIND “Physiopathology and Imaging of Neurological Disorders”, Institut Blood and Brain @ Caen-Normandie, Cyceron, Caen, France.

^2^Department of Biochemistry, CHU de Caen, Caen, France.

^3^GIGA-CRC In Vivo Imaging and Psychology; Cognitive Neuroscience Research Unit, Liège University, and F.R.S-FNRS Belgium.

^4^German Center for Neurodegenerative Disease, Deutsches Zentrum für Neurodegenerative Erkrankungen, DZNE, Dresden, Germany.

^5^Division of Psychiatry, University College London, London, United Kingdom.

^6^Department of Neurology, CHU de Caen, Caen, France.

^7^Department of Clinical Research, Caen Normandy Hospital (CHU) de Caen, Caen, France.

^8^EUCLID/F-CRIN Clinical Trials Platform, INSERM, CHU Bordeaux, University of Bordeaux, Bordeaux, France.

^9^Laboratory for Behavioral Neurology and Imaging of Cognition, Department of Neuroscience, Medical School, University of Geneva, Geneva, Switzerland.

^10^Institut National de la Santé et de la Recherche Médicale, Lyon, France.

^11^Deutsches Zentrum für Neurodegenerative Erkrankungen, Dresden, Germany.
