# Supplementary material for: Allostatic load, a measure of cumulative physiological stress, impairs brain structure but not β-accumulation in older adults: an exploratory study
Source: Front Aging Neurosci. 2025 Mar 31;17:1508677. doi: 10.3389/fnagi.2025.1508677 (PMC11994863; doi:10.3389/fnagi.2025.1508677)
Supplement: Supplementary file 1 [file Data_Sheet_1.DOCX]

# Supplementary Methods

**Table of content:**

**eMethods 1:** Detailed methods for biological measures

**eMethods 2:** Detailed methods for neuroimaging data processing and analyses

**eMethods 3:** Detailed methods for neuropsychological data processing and analyses

**eMethods 1: Detailed methods for biological measures**

Fasting participants’ blood samples were collected by intravenous cannulation into BD Vacutainer tubes (for serum), and into BD K2-EDTA Vacutainer tubes (for plasma) in the morning between 7:30 and 8:30 AM.

**Insulin** *–* The plasma concentration of Insulin was performed by chemiluminescence assay on automated analyzer COBAS 6000 (Roche diagnostics, Meylan, France), using ready-made commercial reagent kits (Insulin ROCHE).

**High-sensitivity C-reactive protein (hsCRP) and Interleukine-6 (IL-6)** *–* The plasma concentration of hsCRP and IL-6 levels were performed by an ultra-sensitive electrochemiluminescence measurement technic (Meso Scale Discovery, MSD, Rockville, Marylan, USA) using V-PLEX^TM^ assays and ready-made reagent kits (V-PLEX Plus Neuroinflammation Panel 1 Human kit, K15210G, MSD).

**Dehydroepiandrosterone Sulfate (DHEA-S)** *–* The serum concentration of DHEA-S was performed by electrochemiluminescence assay (ECLIA) on TECAN EVO (TECAN, Männedorf, Switzerland) using ready-made commercial reagent kits (DHEA-S, ROCHE).

**Cortisol** *–* The serum concentration of cortisol was performed by chemiluminescence assay on automated analyzer COBAS 8000 (Roche diagnostics, Meylan, France) using ready-made commercial reagent kits (Cortisol II, ROCHE).

**Creatinine** *–* The serum creatinine concentration was assessed by an enzymatic method, with a photometric end-point reaction, performed on Beckman Coulter Clinical Chemistry AU analyzers, (Beckman Coulter, Villepinte, France).

**High and low-density cholesterol (HDL, LDL) and Triglycerides** *–* The quantitative determination of total cholesterol, HDL cholesterol and triglycerides concentrations in serum was assessed by an enzymatic staining test in Beckman Coulter Clinical Chemistry AU analyzers (Beckman Coulter, Villepinte, France). LDL cholesterol was calculated according to the Friedewald formula.

**Catecholamines** *–* Norepinephrine (NE) and epinephrine (E) were quantified using liquid chromatography coupled to tandem mass spectrometry (LC-MS/MS). Briefly, catecholamines and their deuterated internal standards (NE-d6 and E-d6, LGC Standards GmbH,Wesel, Germany) were extracted in basic conditions using activated aluminum oxide. After elution in acid condition and evaporation, the analytes were derivatized by sodium cyanoborohydride and acetaldehyde (Merck, Darmstadt, Germany) to form ethyl derivatives as previously reported [1]. Liquid chromatography was conducted on an UFLC chromatographic system (Shimadzu, Kyoto, Japan) connected to a SCIEX QTRAP® 5500 mass spectrometer (SCIEX, Toronto, Canada). Chromatographic separation was performed using a SynergiTM hydro-RP C18 column (Phenomenex, Torrance, USA) in gradient conditions, and mass spectrometry analysis was conducted using the electrospray ion source (ESI) in positive mode. Limit of quantification were <0.01 µg/L for NE and E. Recoveries were 100±7% and 96±5% in plasma samples spiked respectively with NE and E. During reproducibility assay, coefficient of variations (CV) were lower than 8.2% for NE or E at three levels of concentrations for control samples.

**Parasympathetic nervous system functioning** *–* The parasympathetic nervous system markers were calculated based on 2 ECG measures: a standard deviation of the average heart beat-to-beat intervals (SDANN), and root mean square of successive differences between normal heartbeats (RMSSD), recording via 2 sub-clavicular bipolar electrodes in semi recumbent position in the evening and computed by Kubios software (Kubios HRV premium 3.3.1).

**Body mass index (BMI)** *–* BMI was calculated as the ratio between weight in kilograms and height in square meters.

**Waist-hip-ratio (WHR)** *–* WHR was calculated as the ratio between waist circumference in centimeters (cm) and hip circumference in cm.

**Systolic and diastolic blood pressure (SBP, DBP) and pulse pressure** *–* SBP, DBP and pulse pressure were taken using an automatic blood pressure monitor (Omron, M6 Confort). Three different resting blood pressure measurements were averaged to calculate an overall SBP, DBP and pulse pressure score.

**eMethods 2: Detailed methods for** **neuroimaging data processing and analyses**

All participants were scanned on the same magnetic resonance imaging (MRI; Philips Achievia 3.0T scanner) and positron emission imaging (PET; Discovery RX VCT 64 PET-CT scanner, General Electric Healthcare) cameras at the Cyceron Center (Caen, France).

**MRI data:** A high-resolution T1-weighted structural image using a 3D fast-field echo sequence (sagittal; repetition time (RT), 7.1 ms; echo time (ET), 3.3 ms; flip angle, 6°; field of view (FOV), 256 × 256 mm 2 , 180 slices, voxel size: 1 × 1 × 1 mm^3^ ) and a 3D fluid- attenuated inversion recovery (FLAIR; sagittal; RT, 4800 ms; ET, 272 ms; inversion time, 1650 ms; flip angle, 40°; FOV, 250 × 250 mm 2 , 180 slices, voxel size: 0.98 × 0.98 × 1 mm^3^) were acquired. Then, an echo-planar imaging/spin echo diffusion weighted sequence (DKI) was performed at multiple shells: 3b-values (0, 1000, 2000 s/mm^2^) (axial; 30 directions; RT, 6100 ms; ET, 101 ms; flip angle, 90°; FOV, 216 × 216 mm 2, 48 slices, voxel size: 2.7 × 2.7 × 2.7 mm^3^) and additional blips images with b = 0 s/mm 2 (number of signal averages, 9) were acquired in reverse phase encoding direction for susceptibility distortion.

**PET data:** AV45-PET scans were acquired in two separate sessions, with a resolution of 3.76 × 3.76 × 4.9 mm^3^ (FOV, 157 mm). For each PET-scan, 47 plans were obtained with a voxel size of 1.95 × 1.95 × 3.2 mm^3^. Before the PET acquisition, a transmission scan was performed for attenuation correction. For AV45-PET, each participant underwent a 10-min PET scan beginning at the intravenous injection of ~4MBq/Kg of AV45 to measure brain perfusion, and a 10-min PET scan beginning 50 min after the intravenous injection, to measure amyloid deposition. For the FDG-PET scan, participants (n = 92) fasted for at least 6 hours before scanning. After a 30-min resting period in a quiet and dark environment, ~180 MBq of FDG were intravenously injected as a bolus and a 10-min PET acquisition scan was acquired 50 min after injection.

**Image processing:**

T1-weighted MRIs were segmented using FLAIR images and normalized to the Montreal Neurological Institute (MNI) space. GM normalized segments were modulated to correct for nonlinear warping effects using the Statistical Parametric Mapping (SPM12) software’s multiple channels segmentation procedure (<http://www.fil.ion.ucl.ac.uk/spm/software/spm12>).

Raw FLAIR images were coregistered onto their corresponding native space T1-weighted MRI, and WM lesions were segmented by the lesion prediction algorithm LPA [2] implemented in the Lesion Segmentation Toolbox version 2.0.15 ([www.statistical-modelling.de/lst.html](http://www.statistical-modelling.de/lst.html)) for SPM12. A minimum extend threshold of 0.01 cm³ was set. Lesion probability maps were binarized by applying a threshold of 0.5 and lesion masks were thus generated. Lesion masks were then visually inspected and corrected for false positives in corticospinal tracts if necessary, using a specific corticospinal tract mask for each participant, as fully described elsewhere [3].

DKI images were corrected for susceptibility artifacts, eddy current distortions, and subject motion using Functional MRI of the Brain diffusion toolbox (FSL 5.0.9). DKI data were then processed using MATLAB R2012b (MathWorks) and diffusional kurtosis estimator software (DKE, version 2.6) to estimate the diffusional kurtosis tensor [4]. Images were smoothed with a 3.375 × 3.375 × 3.375 mm full-width at half-maximum Gaussian filter. Mean diffusivity (MD) and fractional anisotropy (FA) maps, reflecting WM microstructural integrity [5], were then extracted from the DKE. These maps were coregistered to the T1-weighted MRI and normalized to the MNI template by applying the deformation parameters from the corresponding T1-weighted MRI.

PET data were coregistered onto their corresponding T1-weighted MRI, and normalized using the deformation parameters defined from the T1-weighted procedure. Resulting images were quantitatively normalized using the cerebellar GM as the reference region, resulting in standardized uptake value ratio (SUVR) maps.

The images resulting from the processing procedure described above for each neuroimaging modality were used both to obtain global neuroimaging values and for the voxel-wise analyses, as described below

Averaged global neuroimaging values were obtained by applying a binary mask of GM for preprocessed T1-weighted and PET images, and a binary mask of WM, for preprocessed DKI images. The cerebellum was excluded from both masks, and only voxels with a probability > 60% of being GM and WM were included, to exclude non-GM or WM voxel and reduce the probability of overlap with other tissue classes. For the AV45 values, we used a binary mask corresponding to the entire GM except for the cerebellum, occipital and sensory motor cortices, hippocampi, amygdala, and basal nuclei, as described previously in La Joie et al., 2012.

**eMethods 3: Detailed methods for neuropsychological data processing and analyses**

To determine cognitive normality, participants completed a comprehensive diagnostic battery comprising the following standardized tests:

- Global Cognitive Functioning: Mini-Mental State Examination (MMSE) (Folstein et al., 1975), with scores evaluated against age-, gender-, and education-specific norms.

- Executive Functions: Wisconsin Card Sorting Test (Godefroy & GREFEX, 2009), with a z-score > -1.65 compared to norms adjusted for age, gender, and education.

- Verbal Episodic Memory: RL-RI16 test (Van der Linden et al., 2004), with a z-score > -1.65 compared to norms calculated for age, gender, and education.

- Depressive State: Montgomery and Asberg Depression Rating Scale (Montgomery & Asberg, 1979), with scores < 19 indicating no significant depressive symptoms.

- English Proficiency: Test of Written and Oral Comprehension, with scores < 16/18 used as a criterion.

To obtain robust proxies of cognitive ability and minimize the issue of multiple statistical testing, composite scores were created for the main cognitive domains of interest, instead of using multiple (sub)tests.

The composite measures were chosen based on an extensive review of the literature, followed by a consensus reached during a series of workshops with researchers from the Medit-Ageing European project. Most studies use the PACC (Donohue et al. 2014), PACC5 (Papp et al. 2017), or a variation of these composite scores to reflect global cognition using Z-scores. Additionally, several studies have developed composite scores for specific cognitive domains based on theoretical background (Bangen et al., 2010; Crane et al., 2012; Monsell et al., 2014; Ossenkoppele et al., 2012; Petrou et al., 2012; Pike et al., 2011; Porter et al., 2018; Lim et al., 2016; Sano et al.,2017; Stricker et al., 2011; Tijms et al., 2014).

Subsequently, we decided to create a global cognitive composite score (PACC-5), along with composite scores for processing speed, selective attention, executive functions, and episodic memory, based on the neuropsychological tests available to us. Performance on various cognitive tests were z-transformed and averaged as follows. The composite scores were re-standardised (divided by standard deviation of the baseline data) except for the processing speed and selective attention composite scores. Please note that before averaging, Z-scores derived from reaction times and percentages/number of errors were reversed so that increasing values always indicated a better performance.

- Global cognition composite score; PACC-5 (Preclinical Alzheimer’s Cognitive Composite 5)
- Mattis Dementia Rating Scale-2 (Global score)
- Long-term free recall from the California Verbal Learning Test, second edition (CVLT-II)
- Digit Symbol Substitution Test from the WAIS IV (Raw note)
- Long-term free recall from the Logical Memory Test (Story B) from the WMS IV
- Category Fluency (number of correct Animals recalled in 2 minutes)
- Processing speed composite score
- TMT A (time to perform the Trail Making Test part A)
- Stroop Test, reading time (time to complete the word card, reading condition)
- Stroop Test, naming time (time to complete the colour card, naming condition)
- Selective attention composite score
- Digit Symbol Substitution Test from the WAIS IV (Raw note)
- Number of correct items at the D2-R Test
- Percentage of errors at the D2-R Test
- Executive functions composite score
- Digit Span Backward from the WAIS IV (Raw note)
- TMT B (time to perform the Trail Making Test part B)
- Stroop Test, interference index (time difference between the interference and naming conditions)
- Verbal Fluency (number of correct P-words recalled in 2 minutes)
- Episodic memory composite score
- Sum of trials 1-5 from CVLT-II
- Short-term free recall from CVLT-II
- Long-term free recall from CVLT-II
- Short-term recall from Logical Memory (Story B), WMS IV
- Long-term recall from the Logical Memory (Story B), WMS IV

Abbreviations:

CVLT-II, California Verbal Learning Test - second edition; TMT, Trail Making Test; WAIS, Wechsler Adult Intelligence Scale; WMS, Wechsler Memory Scale.

References:

Donohue, M. C., Sperling, R. A., Salmon, D. P., Rentz, D. M., Raman, R., Thomas, R. G., et al. (2014). The preclinical Alzheimer cognitive composite: measuring amyloid-related decline. JAMA Neurol, 71(8), 961-970, doi:10.1001/jamaneurol.2014.803

Papp, K. V., Rentz, D. M., Orlovsky, I., Sperling, R. A., & Mormino, E. C. (2017). Optimizing the preclinical Alzheimer's cognitive composite with semantic processing: The PACC5. Alzheimers Dement (N Y), 3(4), 668-677, doi:10.1016/j.trci.2017.10.004

Bangen, K. J., Delano-Wood, L., Wierenga, C. E., McCauley, A., Jeste, D. V., Salmon, D. P., & Bondi, M. W. (2010). Associations between stroke risk and cognition in normal aging and Alzheimer’s disease with and without depression. International Journal of Geriatric Psychiatry, 25(2), 175–182. <https://doi.org/10.1002/gps.2317>

Crane, P. K., Carle, A., Gibbons, L. E., Insel, P., Mackin, R. S., Gross, A., Jones, R. N., Mukherjee, S., Curtis, S. M., Harvey, D., Weiner, M., Mungas, D., & for the Alzheimer’s Disease Neuroimaging Initiative. (2012). Development and assessment of a composite score for memory in the Alzheimer’s Disease Neuroimaging Initiative (ADNI). Brain Imaging and Behavior, 6(4), 502–516. <https://doi.org/10.1007/s11682-012-9186-z>

Monsell, S. E., Mock, C., Hassenstab, J., Roe, C. M., Cairns, N. J., Morris, J. C., & Kukull, W. (2014). Neuropsychological changes in asymptomatic persons with Alzheimer disease neuropathology. Neurology, 83(5), 434–440. <https://doi.org/10.1212/WNL.0000000000000650>

Ossenkoppele, R., Zwan, M. D., Tolboom, N., Van Assema, D. M. E., Adriaanse, S. F., Kloet, R. W., &… Van Berckel, B. N. M. (2012). Amyloid burden and metabolic function in early-onset Alzheimer’s disease: Parietal lobe involvement. Brain, 135(7), 2115–2125. <https://doi.org/10.1093/brain/aws113>

Petrou, M., Bohnen, N. I., Müller, M. L. T. M., Koeppe, R. A., Albin, R. L., & Frey, K. A. (2012). Aβ-Amyloid deposition in patients with parkinson disease at risk for development of dementia. Neurology, 79(11), 1161–1167. <https://doi.org/10.1212/WNL.0b013e3182698d4a>

Pike, K. E., Ellis, K. A., Villemagne, V. L., Good, N., Chételat, G., Ames, D., Szoeke, C., Laws, S. M., Verdile, G., Martins, R. N., Masters, C. L., & Rowe, C. C. (2011). Cognition and betaamyloid in preclinical Alzheimer’s disease: Data from the AIBL study. Neuropsychologia, 49(9), 2384–2390. https://doi.org/10.1016/j.neuropsychologia.2011.04.012

Porter, T., Burnham, S. C., Savage, G., Lim, Y. Y., Maruff, P., Milicic, L., Peretti, M., Ames, D., Masters, C. L., Martins, R. N., Rainey-Smith, S., Rowe, C. C., Salvado, O., Taddei, K., Groth, D., Verdile, G., Villemagne, V. L., & Laws, S. M. (2018). A Polygenic Risk Score Derived From Episodic Memory Weighted Genetic Variants Is Associated With Cognitive Decline in Preclinical Alzheimer’s Disease. Frontiers in Aging Neuroscience, 10. <https://doi.org/10.3389/fnagi.2018.00423>

Lim, Y. Y., Snyder, P. J., Pietrzak, R. H., Ukiqi, A., Villemagne, V. L., Ames, D., Salvado, O., Bourgeat, P., Martins, R. N., Masters, C. L., Rowe, C. C., & Maruff, P. (2016). Sensitivity of composite scores to amyloid burden in preclinical Alzheimer’s disease: Introducing the Zscores of Attention, Verbal fluency, and Episodic memory for Nondemented older adults composite score. Alzheimer’s & Dementia: Diagnosis, Assessment & Disease Monitoring, 2, 19–26. <https://doi.org/10.1016/j.dadm.2015.11.003>

Sano, M., Zhu, C. W., Grossman, H., & Schimming, C. (2017). Longitudinal Cognitive Profiles in Diabetes: Results From the National Alzheimer’s Coordinating Center’s Uniform Data. Journal of the American Geriatrics Society, 65(10), 2198–2204. <https://doi.org/10.1111/jgs.15014>

Stricker, N. H., Chang, Y. L., Fennema-Notestine, C., Delano-Wood, L., Salmon, D. P., Bondi, M. W., & Dale, A. M. (2011). Distinct profiles of brain and cognitive changes in the very old with Alzheimer disease. Neurology, 77(8), 713–721. <https://doi.org/10.1212/WNL.0b013e31822b0004>

Tijms, B. M., Yeung, H. M., Sikkes, S. A. M., Möller, C., Smits, L. L., Stam, C. J., … Barkhof, F. (2014). Single-subject gray matter graph properties and their relationship with cognitive impairment in early- and late-onset Alzheimer’s disease. Brain Connectivity, 4(5), 337–346. <https://doi.org/10.1089/brain.2013.0209>

**Supplementary Tables**

**eTable 1:** Characteristics of studies investigating the association between AL and brain integrity.

**eTable 2:** Multiple linear regressions between each AL category and global neuroimaging values adjusted for age, sex, education, smoking status and current medication use.

**eTable 3:** Multiple linear regressions between each AL category and cognitive composite scores adjusted for age, sex, education, smoking status and current medication use.

**eTable 4:** Associations between primary mediators (neuroendocrine and immune categories) and other AL categories.

**eTable 1: Characteristics of studies investigating the association between AL and brain integrity.**

| **Study** | **Sample size** | **AL biomarkers** | **AL calculation method** | **System: biomarkers** | **Brain outcome assessed** | **Covariates** | **Significant results – association with AL** |
| --- | --- | --- | --- | --- | --- | --- | --- |
| Booth et al. (2015) | N=658  Mean age: 72.5 years  48% female | 10 | Factor analysis | Immune: Fib, CRP, IL-6  Metabolic: TG, HDL, LDL, HbA1c, BMI  Cardiovascular: SBP, DBP  Neuroendocrine: *None* | Grey , and white matter volumes | Age, sex, education, socioeconomic status, intellectual quotient at 11 years old | Negative association with white matter volume |
| Ritchie et al. (2017) | N=731  Mean age: 72.68 years  46.9% female | 8 | *Not specified* | Immune: Fib, CRP, IL-6  Metabolic: TG, HDL, LDL, HbA1c, BMI  Cardiovascular: *None*  Neuroendocrine: *None* | Grey , white matter volumes, and white matter hyperintensities  Fractional anisotropy and mean diffusivity | Age, sex | Negative association with grey , white matter volumes and fractional anisotropy  Positive association with mean diffusivity |
| Zsoldos et al. (2018) | N=349  Mean age: 69.6 years  19.5% female | 9 | High-risk cut-off method | Immune: CRP, IL-6  Metabolic: insulin, glycemia, TG, HDL, LDL  Cardiovascular: SBP, DBP  Neuroendocrine: *None* | Grey matter volume  Fractional anisotropy and mean diffusivity | Age, sex, education, ethnicity, employment grade | Negative association with grey matter volume |

*Abbreviations: BMI, Body mass index; CRP, C-reactive protein; Fib, Fibrinogen; HDL and LDL, High- and Low-density lipoprotein; HbA1c, Glycated hemoglobin; IL-6, Interleukin-6; SBP and DBP, Systolic and Diastolic blood pressure; TG, Triglycerides.*

1. Ji C, Walton J, Su Y *et al.* Simultaneous determination of plasma epinephrine and norepinephrine using an integrated strategy of a fully automated protein precipitation technique, reductive ethylation labeling and UPLC-MS/MS. *Anal Chim Acta* 2010;**670**:84–91.

2. Schmidt P. Bayesian inference for structured additive regression models for large-scale problems with applications to medical imaging. Dissertation, LMU München: Faculty of Mathematics, Computer Science and Statistics. 2016:Chapter 6.1.

3. Garnier-Crussard A, Bougacha S, Wirth M *et al.* White matter hyperintensities across the adult lifespan: Relation to age, Aβ load, and cognition. *Alzheimer’s Res Ther* 2020;**12**:1–11.

4. Tabesh A, Jensen JH, Ardekani BA *et al.* Estimation of tensors and tensor-derived measures in diffusional kurtosis imaging. *Magn Reson Med* 2011;**65**:823–36.

5. Falangola MF, Jensen JH, Babb JS *et al.* Age-related non-Gaussian diffusion patterns in the prefrontal brain. *J Magn Reson Imaging* 2008;**28**:1345–50.

**eTable 2: Multiple linear regressions between each AL category and global neuroimaging values adjusted for age, sex, education, smoking status and current medication use.**

|  | **Neuroendocrine** | **Immune** | **Metabolic** | **Cardiorespiratory** | **Anthropometric** |
| --- | --- | --- | --- | --- | --- |
| **GMV** | β, .14  [95% CI, -5.7^e^-6 ; 8.84^e^-6]  P=.68 | β, -.15  [95% CI, -.00 ; 2.66^e^-6]  P=.20 | β, -.33  [95% CI, -.00 ; .1-7^e^-7]  P=.04 | β, -.01  [95% CI, -7.52^e^-6 ; 8.00^e^-6]  P=.95 | β, -.51  [95% CI, -2.13^e^-5 ; -8.34^e^-6]  **P<.001** |
| **Glucose metabolism** | β, -.07  [95% CI, -5.99 ; 1.67]  P=.26 | β, .16  [95% CI, -.26 ; 2.74]  P=.10 | β, .03  [95% CI, -1.04 ; 5.28]  P=.18 | β, -.03  [95% CI, -4.04 ; 3.40]  P=.86 | β, .06  [95% CI, -1.73 ; 5.03]  P=.33 |
| **Brain perfusion** | β, -.04  [95% CI, -7.27 ; 1.89]  P=.23 | β, -.13  [95% CI, -8.27 ; 2.04]  P=.23 | β, .00  [95% CI, -2.76 ; 6.33]  P=.43 | β, -.05  [95% CI, -6.33 ; 4.04]  P=.66 | β, -.07  [95% CI, -4.56 ; 4.87]  P=.95 |
| **Amyloid deposition** | β, .06  [95% CI, -.73 ;2.08]  P=.34 | β, -.07  [95% CI, -2.10 ;.87]  P=.41 | β, .10  [95% CI, -.92 ;1.68]  P=.57 | β, -.19  [95% CI, -3.02 ; -.08]  P=.04 | β, .11  [95% CI, -.82 ;1.91]  P=.43 |
| **WM Hyper-intensities** | β, .13  [95% CI, -.02 ;.06]  P=.45 | β, .04  [95% CI, -.03 ; .05]  P=.81 | β, .11  [95% CI, -.02 ; .05]  P=.46 | β, -.06  [95% CI, -.06 ; .03]  P=.54 | β, .02  [95% CI, -.04 ; .04]  P=.86 |
| **MD** | β, -.03  [95% CI, -2.44 ; 3.99]  P=.63 | β, .16  [95% CI, -1.17 ; 5.59]  P=.20 | β, .26  [95% CI, -2.4 ; 3.49]  P=.74 | β, .11  [95% CI, -2.03 ; 4.79]  P=.42 | β, .40  [95% CI, 1.50 ; 7.51]  **P=.003** |
| **FA** | β, -.24  [95% CI, -13.84 ; 5.83]  P=.42 | β, -.05  [95% CI, -17.59 ; 3.10]  P=.17 | β, -.15  [95% CI, -8.41 ; -.88]  p=.87 | β, -.08  [95% CI, -14.34 ; 6.55]  P=.46 | β, -.29  [95% CI, -21.43 ; -2.89]  P=.01 |

*Results are displayed with unstandardized β, [95% CI], P-value. All analyses were adjusted for age, sex, education, smoking status and current medication use. Note: Bonferroni correction was applied to control for multiple comparisons so that a threshold of P < .00714 (.05/7) was required for results to be considered significant (in bold). Abbreviations: FA, fractional anisotropy; GMV, gray matter volume; MD, mean diffusivity; WM, white matter.*

**eTable 3: Multiple linear regressions between each AL category and cognitive composite scores adjusted for age, sex, education, smoking status and current medication use.**

|  | **Neuroendocrine** | **Immune** | **Metabolic** | **Cardiorespiratory** | **Anthropometric** |
| --- | --- | --- | --- | --- | --- |
| **PACC-5** | β, .07  [95% CI, -.22 ; .19]  P= .89 | β, -.06  [95% CI, -.26 ; .18]  P= .74 | β, -.16  [95% CI, -.20 ; .19]  P= .94 | β, -.06  [95% CI, -.27 ; .18]  P= .67 | β, -.05  [95% CI, -.11 : .30]  P= .36 |
| **Processing speed** | β, -.06  [95% CI, -.33 ; .18]  P= .57 | β, .09  [95% CI, -0.13; 0.40]  P= .33 | β, .05  [95% CI, -.15 ; .32]  P= .49 | β, .10  [95% CI, -.13 ; .41]  P= .31 | β, -.04  [95% CI, -.31 ; .19]  P= .65 |
| **Selective attention** | β, .04  [95% CI, -.29 ; .26]  P= .91 | β, .07  [95% CI, -.15 ; .43]  P= .35 | β, .11  [95% CI, .07 ; .57]  P= .01 | β, .20  [95% CI, .01 ; .59]  P= .04 | β, .14  [95% CI, .07 ; .59]  P= .01 |
| **Executive functions** | β, -.05  [95% CI, -.52 ; .07]  P= .14 | β, -.30  [95% CI, -.74 ; -.14]  **P= .005** | β, -.02  [95% CI, -.18 ; .37]  p= .53 | β, -.07  [95% CI, -.41 ; .21]  p= .52 | β, -.08  [95% CI, -.26 ; .32]  P= .82 |
| **Episodic memory** | β, .18  [95% CI, -.14 ; .28]  P= .52 | β, -.10  [95% CI, -.32 ; .13]  P= .39 | β, -.19  [95% CI, -.24 ; .15]  P= .60 | β, .02  [95% CI, -.18 ; .27]  P= .67 | β, -.13  [95% CI, -.18 ; .23]  P= .80 |

*Results are displayed with unstandardized β, [95% CI], P-value. All analyses were adjusted for age, sex, education, smoking status and current medication use. Note: Bonferroni correction was applied to control for multiple comparisons so that a threshold of P < .01 (.05/5) was required for results to be considered significant (in bold). Abbreviation: PACC-5; Preclinical Alzheimer’s Cognitive Composite 5.*

**eTable 4: Associations between primary mediators (neuroendocrine and immune categories) and other AL categories.**

|  | **Neuroendocrine category** | **Immune category** |
| --- | --- | --- |
| Metabolic category | β, -.25 [95% CI, -.46;-.05], **P = .01** | β, .09 [95% CI, -.13; .31], P = .42 |
| Cardiorespiratory category | β, .001 [95% CI, -.18; .18], P = .99 | β, .06 [95% CI, -.13; .25], P = .54 |
| Anthropometric category | β, -.21 [95% CI, -.41;-.01], P = .03 | β, .158 [95% CI, -.05; .37], P = .14 |

*Results are displayed with unstandardized β, [95% CI], P-value. All analyses were adjusted for age, sex, education, smoking status and current medication use. Note: Bonferroni correction was applied to control for multiple comparisons so that a threshold of P < .016 (.05/3) was required for results to be considered significant (in bold).*
